# Segmentation of Choroidal Boundary in Enhanced Depth Imaging OCTs Using a Multiresolution Texture Based Modeling in Graph Cuts

**DOI:** 10.1155/2014/479268

**Published:** 2014-02-11

**Authors:** Hajar Danesh, Raheleh Kafieh, Hossein Rabbani, Fedra Hajizadeh

**Affiliations:** ^1^Department of Biomedical Engineering, Medical Image and Signal Processing Research Center, Isfahan University of Medical Sciences, Isfahan 81745, Iran; ^2^Noor Ophthalmology Research Center, Tehran 1968653111, Iran

## Abstract

The introduction of enhanced depth imaging optical coherence tomography (EDI-OCT) has provided the advantage of in vivo cross-sectional imaging of the choroid, similar to the retina, with standard commercially available spectral domain (SD) OCT machines. A texture-based algorithm is introduced in this paper for fully automatic segmentation of choroidal images obtained from an EDI system of Heidelberg 3D OCT Spectralis. Dynamic programming is utilized to determine the location of the retinal pigment epithelium (RPE). Bruch's membrane (BM) (the blood-retina barrier which separates the RPE cells of the retina from the choroid) can be segmented by searching for the pixels with the biggest gradient value below the RPE. Furthermore, a novel method is proposed to segment the choroid-sclera interface (CSI), which employs the wavelet based features to construct a Gaussian mixture model (GMM). The model is then used in a graph cut for segmentation of the choroidal boundary. The proposed algorithm is tested on 100 EDI OCTs and is compared with manual segmentation. The results showed an unsigned error of 2.48 ± 0.32 pixels for BM extraction and 9.79 ± 3.29 pixels for choroid detection. It implies significant improvement of the proposed method over other approaches like *k*-means and graph cut methods.

## 1. Introduction

Optical coherence tomography (OCT) imaging technique was introduced by Huang et al. in 1991 [1]. This technique is employed for its ability in taking cross-sectional images from microscopic structure of the living tissues. The device has a resolution on the scale of micrometers. In this technique, a number of A-scans or linear scans create B-scan or cross-sectional images [2]. OCT images consist of a great amount of data; therefore, nonautomated and visual analysis of such a great amount of data would be troublesome for the ophthalmologist. The main goal of automatic segmentation is to assist the ophthalmologist in diagnosis and monitoring of eye diseases.

**Figure 1:**
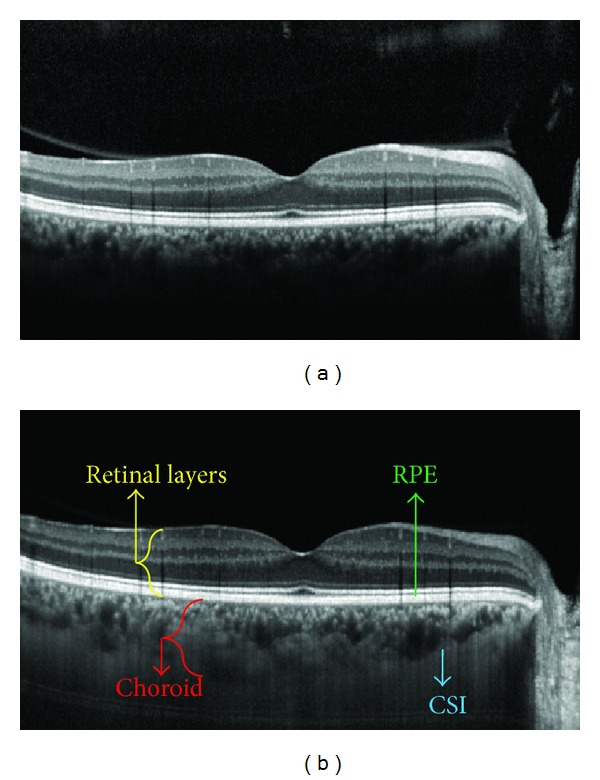
An example of EDI OCT imagining (b), compared to conventional OCT (a).

Choroid is one of the structural layers located between the sclera and the retina. This pigmented layer contains many capillaries that supply feeding of the iris and retinal light receptor cells [3]. Many diseases such as polypoidal choroidal vasculopathy and choroidal tumors cause changes in the structure of this layer [4–8]; therefore, segmentation of this layer has great importance for ophthalmologists. In retinal imaging using conventional OCT, wavelength of the light source is around 800 nanometers, which is not appropriate for imaging of choroid layer, due to signal transmission difficulty through retinal pigment epithelium (RPE) layer and increased depth of imaging [9]. However, increased pixel density and high signal-to-noise ratio in SD-OCT (spectral-domain OCT) in comparison to TD-OCT (time-domain OCT) makes choroidal imaging possible. In SD-OCT, the structures that are closer to “zero delay line” have higher signals than those that are farther away. In conventional OCT, the “zero delay line” is near the inner surface of retina; however, in EDI-OCT, it is placed near the outer retina and choroid, which is the key for EDI imaging. In order to increase the quality of EDI-OCT and to reduce “speckles,” a high number of images (usually 100 images) are averaged through a software that provides high-quality images with smoother border [9]. An example of this imaging method is compared with conventional OCT in Figure 1. However, another solution for choroidal imaging is a higher wavelength of approximately 1060 nanometer [10, 11].

In several studies, EDI-OCT has been used to measure the thickness of the choroid, finding its relation with diseases, and monitoring the treatment process [12–14]. In most of these studies, measurement of choroidal thickness is usually accomplished by manual labeling which is a time-consuming and tedious process. This problem is much more complicated when the number of images is numerous. Therefore, the need for development of an automatic segmentation algorithm on EDI-OCT arises.

A limited number of studies have already been conducted about automatic segmentation of these images. Kajić et al. [15–17] proposed a two-stage statistical model to detect the choroid boundaries in the 1060 nm OCT images in healthy and pathological eyes. This model needed extensive training and the mean error is 13%. Tian et al. [18, 19] found the choroidal boundary by finding the shortest path of the graph formed by valley pixels using dynamic programming (DP). The average of Dice's coefficient on 45 EDI-OCT images was 90.5%. Lu et al. [20] proposed a technique to segment the inner boundary of the choroid using a two-stage fast active contour model. Then a real-time human-supervised automated segmentation on the outer boundary of the choroid was applied. The reported Dice similarity coefficient value on 30 images captured from patients diagnosed with diabetes was 92.7%.

Many algorithms based on wavelet and graph cut are already used in segmentation of retinal layers [21]. However, due to heterogeneity in the choroid layers, such methods cannot be useful in choroid segmentation. However, because of distinct difference between texture of choroid and other retinal layers, an algorithm based on texture classification can be effective. With this idea, we use a combination of graph-based methods and wavelet-domain features for choroidal segmentation. A description of our method including detection of Bruch's membrane (BM) and segmentation of choroidal-scleral interface (CSI) is provided in Section 2 and the results are presented in Section 3. The results show the improved robustness of the method compared to other available algorithms. Finally the conclusion and suggestions for future works are presented in Section 4.

## 2. Material and Methods

The proposed choroid segmentation method is tested on 100 randomly selected two-dimensional EDI-OCT images obtained from 10 eyes of 6 normal subjects by an EDI system of multimodality diagnostic imaging (wavelength: 870 nm; scan pattern: enhanced depth imaging; Spectralis HRA + OCT; Heidelberg Engineering, Heidelberg, Germany) [22]. The study protocol was reviewed in advance by the review board of Noor Ophthalmology Research Center. Each participant was informed of the purpose of the study and provided a written consent to participate. For evaluation and ruling out any ocular pathology all patients underwent thorough ophthalmic examinations including refraction, visual acuity, slit lamp biomicroscopic examination, IOP measurement by Goldmann applanation tonometer, and examination of the fundus with plus 90-D lens. Each dataset consisted of a SLO image and limited number of OCT scans with size of 496 × 768 (namely, for a data with 31 selected OCT slices, the whole data size would be 496 × 768 × 31). The pixel resolutions were 3.9 *μ*m/pixel in axial direction and 14 *μ*m/pixel in transversal direction.

### 2.1. Overview of Algorithm

In the first step, dynamic programming (DP) was applied for boundary detection of RPE layer. The BM is the blood-retina barrier that separates the RPE cells of the retina from the choroid and can be segmented by searching for the pixels with the biggest gradient value below the RPE. In this stage, we eliminated the pixels above this boundary form our calculations by making them equal to zero and focused on segmentation of the lower boundary of the choroid.

**Figure 2:**
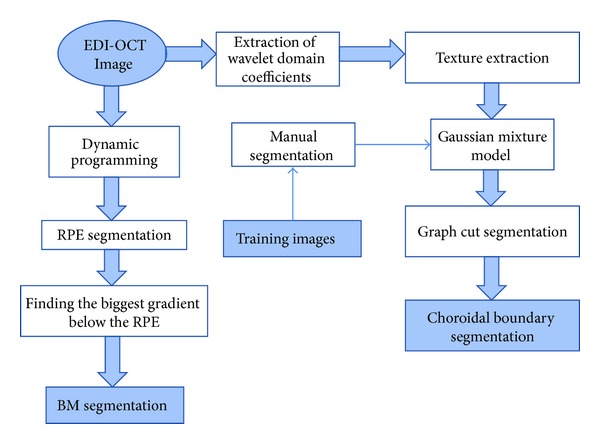
Choroid segmentation algorithm overview.

In the next step, discrete wavelet transform (DWT)—using filter bank and descriptors—was employed to extract appropriate features from EDI-OCT images. The extracted features from training images were used to construct a Gaussian mixture model (GMM). It is assumed that each extracted descriptor using DWT has a multivariate normal probability density function (pdf) and the number of Gaussian distributions (*K*) is chosen to be 3, which represent the area above the BM, choroid, and the area beneath the CSI. Finally, the relevance of each data to the model is used in creating the graph for final CSI segmentation. A block diagram of the algorithm could be found in Figure 2.

### 2.2. BM Detection

We applied boundary detection algorithm using DP to detect boundary of RPE layer which is the brightest part in EDI OCT images. DP is a method based on optimality [23], which seeks for the best functions in which the variables are not simultaneously in conjunction with each other. In simple problem of boundary tracking, the goal is to find the best path (least expensive) between one of the possible starting points and one of the possible ending points. In this step, upper bound of choroid (lower bound of RPE) can be segmented.

**Figure 3:**
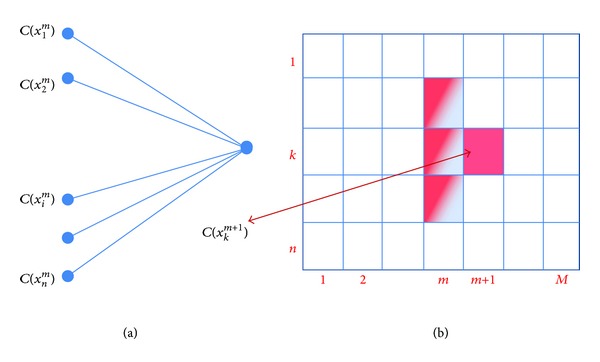
Dynamic programming: (a) one step of cost calculation; (b) graph layers and nodes notation. The highlighted pixels show the neighbors.

For boundary detection using DP, we are looking for a dark boundary transmitted from one edge to another edge in an image (e.g., from column 1 to *M* − 1 in Figure 3). DP algorithm associates a cost to each node (brightness of each pixel) and the partial paths are set according to the cost (brightness) of the next neighboring node. The algorithm starts through the columns (column 1 to *M* − 1) and for each node (from 1 to *n*), the best path with optimized (lowest) cost is selected among three neighbor pixels of the neighboring column (*i* = −1,0, 1 in (1)). Therefore, a cumulative cost (*C*(*x*_*k*_^*m*+1^)) can be made for a pixel in *m* + 1th column and in the *k*th node, according to the cost of previously selected nodes (1).

The following algorithm describes the DP method [24].

(1) Specify initial costs *C*⁡(*x*_*i*_^1^) of all nodes in the first graph layer, *i* = 1,…, *n* and partial path costs *g* ^*m*^(*i*, *k*), *m* = 1,…, *M* − 1.
(2) Repeat step 3 for all *m* = 1,…, *M* − 1.
(3) Repeat step 4 for all nodes *k* = 1,…, *n* in the graph layer *m*.
(4) Let
  (1) $$\begin{array}{c} C\left(x_{k}^{m+1}\right)=\;\underset{i=-1,0,1}{\min}(C(x_{k+i}^{m})+g^{m}\left(i,k\right)). \end{array}$$
  Set pointer from node *x*_*k*_^*m*+1^ back to node *x*_*k*_^*m*∗^, where ∗ denotes the optimal predecessor. Optimality is defined as minimization of the cost function.
(5) Find an optimal node *x*_*k*_^*M*∗^ in the last graph layer *M* and obtain an optimal path by backtracking through the points from *x*_*k*_^*M*∗^ to *x*_*k*_^1∗^.

The positions of a sample node *C*(*x*_*k*_^*m*+1^) and its neighbors in the previous (*m*th) graph layer are shown in Figures 3(a) and 3(b). The highlighted pixels around *C*(*x*_*k*_^*m*+1^) indicate *i* = −1,0, 1 in (1).

**Figure 4:**
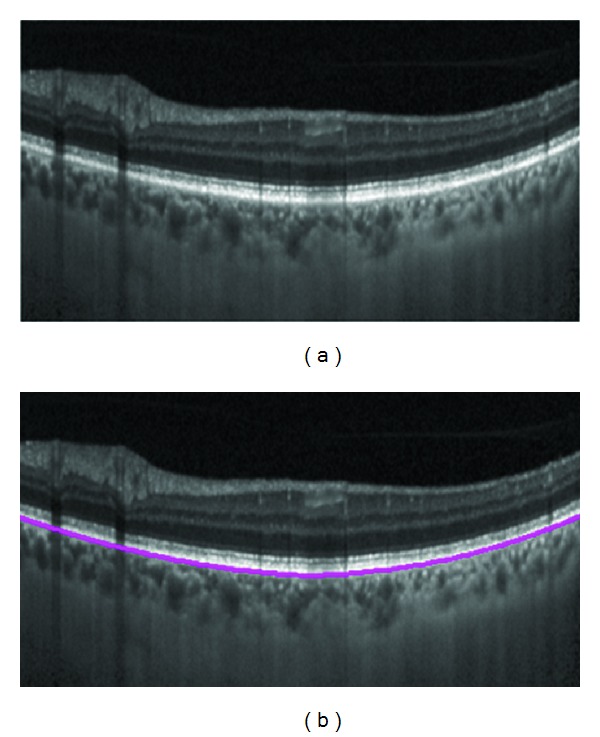
(a) EDI-OCT image of the human eye. (b) Bruch's membrane.

It should also be noted that the DP boundary detection is conventionally looking for the darkest boundary in an image and for our application (detection of the brightest boundary) we should inverse the brightness values of the image. After segmentation of RPE layer, BM can be located by searching for the pixels with the biggest gradient below the RPE.  Figure 4 shows the result of BM segmentation for a sample EDI-OCT image. From calculative point of view, DP is efficient and flexible and can be considered as a powerful tool even in presence of noise.

### 2.3. CSI Detection

A novel combination of algorithms is proposed for discrimination of lower boundary of choroid (CSI). Among many possible texture descriptors used for segmentation of images with several textures [24], we show the ability of wavelet texture descriptors in this work.

The algorithm can then be stated in three steps:

1. calculate wavelet descriptors for each pixel (Section 2.3.1);
2. create a model of the classes using training images. (Section 2.3.2);
3. take a new image and segment it using the learned model. Use graph cut segmentation to obtain spatially coherent segmentation (Section 2.3.3).

It is assumed that extracted descriptors using DWT have a multivariate normal pdf. This assumption can be justified by the central limit theorem (CLT). In its classical form, the CLT states that the mean of *n* independent identically distributed random variables, with finite mean and variance, has a limiting distribution for *n* → *∞* that is Gaussian. As an approximation, for a finite number of observations (*n*⋘*∞*), it provides a reasonable approximation only close to the peak of the normal distribution rather than stretching into the tails (convergence to the limit).

In our datasets of Heidelberg 3D OCT Spectralis, each image is calculated from the mean value of 100 images taken at the same time. The mean process dramatically improves the quality of the information in the images, without introducing an alteration to the original image or adding any distracting electronic noise [22]. Therefore, according to the “convergence to the limit” theorem near the peak areas (needed for GMM assumption), and in accord with linear characteristics of the wavelet transform, we can assume that each extracted descriptor using DWT has a multivariate normal pdf.

#### 2.3.1. Wavelet Descriptors

DWT using filter bank and descriptors is employed to extract features for each pixel of the image. In other applications of wavelets transform for texture analysis, most prevalent features are wavelet energy signatures and their second-order statistics [25, 26]. In this case, Haar wavelet filters (*H*(*z*) = (1 + *z*)/2 and *G*(*z*) = (*z* − 1)/2) are utilized for ease of use. Filter bank stages are repeated (multiresolution approach) for four levels. Since we are using wavelet frame instead of the standard DWT, the number of levels can optionally grow. But, with trial and error, we found that, for levels higher than 4, the results did not improve and we only faced with more complexity. The energy of each high-pass filter at different stages of subbands and the energy of the last stage of the low-pass filter are extracted as image features (5 features). Extracted features and a training image with a known segmentation (mask) are then used to construct GMM for the image.

#### 2.3.2. Gaussian Mixture Model

GMM is a parametric pdf represented as a weighted sum of Gaussian component distributions. The idea for clustering with GMMs is the same as for *k*-means. In conventional GMM, we make an initial guess for mean *μ*_*k*_ and covariance Γ_*k*_ and then try to iteratively refine them. The number of Gaussian distributions (*K*) is chosen to be 3, which represent the area above the BM, choroid, and the area beneath CSI.

Assume that *n*-dimensional data constituted *K* Gaussian distribution, *ℵ*_1_, *ℵ*_2_,…, *ℵ*_*k*_, in which *ℵ*_*k*_ with the mean (*μ*_*k*_) and covariance Γ_*k*_ is considered as follows:

(2) $$\begin{array}{c} {ℵ}_{k}\sim N\left(\mu_{k},\Gamma_{k}\right). \end{array}$$

Considering the weights of each relevant *K* as  *π*_*k*_, (∑_*k*=1_^*K*^ *π*_*k*_ = 1), the final pdf is defined as follows:

(3) $$\begin{array}{c} p\left(x_{j}\right)=\sum_{k=1}^{K}\pi_{k}p\left(x_{j}\;|\;{ℵ}_{k}\right), \end{array}$$

in which

(4) p(xj ∣ ℵk) =1(2π)n/2|Γk|1/2exp⁡(−12(xj−μk)TΓk−1(xj−μk)).

Since we are working on wavelet features described in Section 2.3.1, *n* should be set to 5. We now assume that actual values of the observed EDI-OCT images *X* = {*X*_1_, *X*_2_,…, *X*_*i*_,…*X*_*M*_} and value of pixels in each image *X*_1_ = {*x*_1_, *x*_2_,…, *x*_*j*_,…*x*_*A*×*B*_} are independent. We also suppose that EDI images have equal size of *A* × *B*. With defining *N* = *M* × *A* × *B*, we will have the following equations:

(5) p(X)=∏j=1Np(xj)=∏j=1N∑k=1kπk(2π)n/2|Γk|1/2 ×exp⁡(−12(xj−μk)TΓk−1(xj−μk)),

where *M* represents the number of EDI-OCT images used for training (10 for our case). For each training image, a mask was made manually which classifies each image into three labels: the area above the BM, choroid, and the area beneath CSI.

In conventional GMM, the parameters are estimated using iterative EM algorithm to fit a mixture model to a set of available data [24, 27].

EM algorithm is rapidly repeated and at each stage, Gaussian expectation is estimated for each data sample, and then Gaussian element estimation (maximization) is altered. If *μ*_*k*_ and Γ_*k*_ are in hand as an estimation, we can calculate the *k*th Gaussian probability for *x*_*j*_:

(6) $$\begin{array}{c} p_{jk}=\frac{\pi_{k}p\left(x_{j}\;|\;{ℵ}_{k}\right)}{\sum_{i=1}^{k}\pi_{i}p\left(x_{j}\;|\;{ℵ}_{i}\right)}, \end{array}$$

in which *x*_*j*_ (probability ratio for *ℵ*_*k*_) is granted based on sum of *x*_*j*_ (regardless of Gaussian production) balanced with *π*_*i*_. Therefore, the following equation is defined:

(7) $$\begin{array}{c} \pi_{k}^{\text{new}}=\frac{1}{N}\sum_{j=1}^{N}p_{jk}. \end{array}$$

In this equation, mean *p*_*jk*_ is calculated in data series. Similarly, one can estimate corrected values of *μ*_*k*_ and Γ_*k*_

(8) $$\begin{array}{c} \mu_{k}^{\text{new}}=\frac{\sum_{j=1}^{N}p_{jk}x_{j}}{\sum_{j=1}^{N}p_{jk}},\\ \Gamma_{k}^{\text{new}}=\frac{\sum_{j=1}^{N}p_{jk}\left(x_{j}-\mu_{k}^{\text{new}}\right){\left(x_{j}-\mu_{k}^{\text{new}}\right)}^{T}}{\sum_{j=1}^{N}p_{jk}}. \end{array}$$

With a slight modification to conventional GMM, which uses EM algorithm to calculate the parameters of each Gaussian function (including mean and covariance in (8)), we use the training step for finding the parameters of GMM. Namely, we use training images with known segmentation (mask) and calculate the mean and covariance of each section (for the training data). These parameters are used for all images in our database (i.e., there is no need to use EM algorithm for each image separately, which speeds up the algorithm). Then we only calculate the responsibility factor by (6) to construct a learned model to be fed to graph cut segmentation.

When a new test image is considered for segmentation, we calculate wavelet descriptors for each pixel (and accordingly, 5-dimensional vectors of *x*_*j*_ and *μ*_*k*_). The probability (*p*) of a pixel belonging to a particular class of the learned model can be obtained, afterward. The value of probability (*p*) is then used in the construction of a graph cut segmentation.

#### 2.3.3. Graph Cut Segmentation

Graph cut segmentation is constructed based on a learned model to obtain spatially coherent segmentation.

The direct use of optimization algorithms of minimum-cut/maximum flow for graph partitioning was first introduced by Greig et al. [28] in image processing of binary images. Using graph optimization algorithms, a powerful method of optimal bordering and region classification in *N*-dimensional image data was proposed [29, 30].

This method starts by one or more points representing “object” and one or more points representing “background,” determined using interactive or automatic identification. The overall shape of cost function *C* is represented as follows [31]:

(9) $$\begin{array}{c} C\left(f\right)=c_{\text{data}}\left(f\right)+c_{\text{smooth}}\left(f\right). \end{array}$$

**Figure 5:**
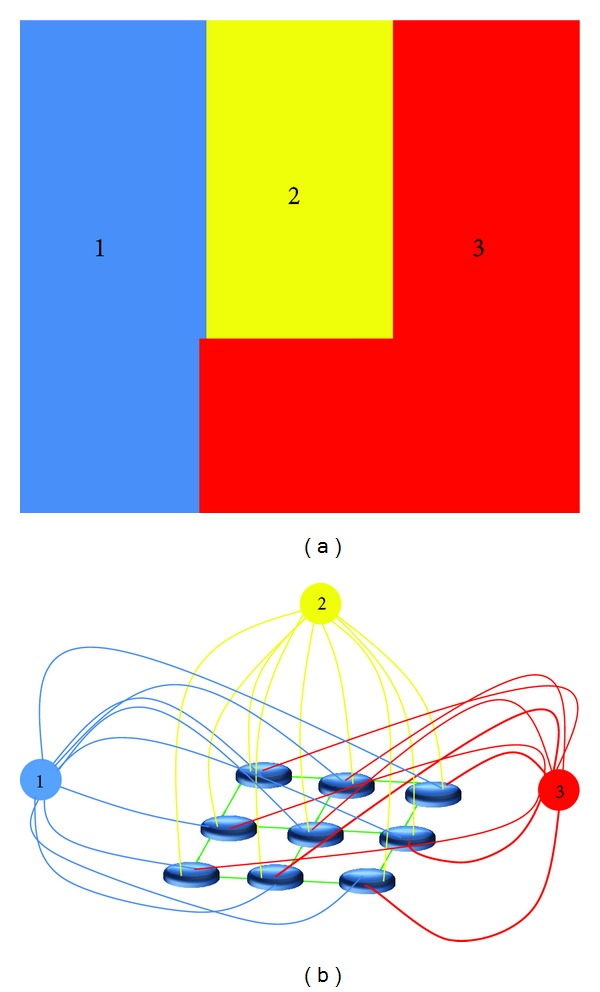
Graph cut classification—example of simple classification. (a) Image with 3 classes. (b) Related graph and terminal nodes. Green lines show *n*-links, while red, yellow, and blue curves represent the *t*-links.

To minimize *C*(*f*), a special class of arc-weighted graphs *G*_*st*_ = (*V* ∪ {terminal  nodes}, *E*) is employed. In addition to the set of nodes *V* corresponding to pixels of the image *I* (Figure 5(a)), terminal nodes (shown by 1, 2, and 3 in Figure 5(b)) are also added to *G*_*st*_. These terminals are hard-linked with the segmentation seed points (bold links in Figure 5(b)) and represent the segmentation labels (1, 2, and 3).

The arcs *E* in *G*_*st*_ can be classified into two categories: *n*-links and *t*-links. The *n*-links connect pairs of neighboring pixels whose costs are derived from the smoothness term *c*_smooth_(*f*). The *t*-links connect pixels whose costs are derived from the data term *c*_data_(*f*). Green lines in Figure 5(b) show *n*-links, while red, yellow, and blue curves represent the *t*-links. A *s* − *t* cut in *G*_*st*_ is a set of arcs whose removal partitions the nodes into three disjoint subsets (1, 2, and 3 in Figure 5). The cost of a cut is the total cost of arcs in the cut, and a minimum *s* − *t* cut is a cut whose cost is minimal. The minimum *s* − *t* cut problem or its dual (the maximum flow problem) can be solved by various algorithms. In maximum flow algorithm, maximum amount of water to sink is delivered using directed arc graphs and the amount of water flow through each separate arc is determined using arc capacity or cost. The greatest amount of maximum flow from *s* to *t* saturates a set of graph arcs. These saturated arcs divide nodes into two separate sections of *S* and *T*, related to minimum cuts [24, 32, 33].

Let each image pixel *i*_*k*_ take a binary label *L*_*k*_ ∈ {1,2, 3}. The labeling vector *L* = (*L*_1_, *L*_2_,…, *L*_|*I*|_) defines the resulting binary segmentation.

In this paper, *C*_data_(*f*) is the distance between each image pixel and initial class made by GMM in Section 2.3.2. For a test image, wavelet descriptors are calculated for each pixel and probability (*p*) of a pixel belonging to a particular class of the learned model is obtained. Then *C*_data_(*f*) is created by

(10) $$\begin{array}{c} C_{\text{data}}\left(f\right)=-\log\left(p+eps\right). \end{array}$$

*C*_smooth_(*f*) is a matrix of costs which is related to adjacent pixel values. In this work, we assigned a square matrix with fixed values (*r* = 4) and with zeros on diagonal as *C*_smooth_(*f*) (9). The increase in fixed term (*r*) would enhance adjacency constraint constant and therefore would result in a finer separation:

(11) $$\begin{array}{c} C_{\text{smooth}}\left(f\right)=\left[\begin{array}{cccccc} 0 & r & r & \cdots & \cdots & r\\ r & 0 & r & r & \ddots & \vdots \\ r & r & 0 & r & r & \vdots \\ \vdots & r & r & 0 & r & r\\ \vdots & \ddots & r & r & 0 & r\\ r & \ldots & \ldots & r & r & 0 \end{array}\right]. \end{array}$$

## 3. Results

One hundred two-dimensional EDI-OCT images were obtained from Heidelberg 3D OCT Spectralis and were used in statistical analysis to produce the results. For our 100 two-dimensional dataset, we chose 10 images to train the learned model. Actually, we can choose any 10 or less images for training and calculate means and covariances and weights of GMM and there would be no considerable difference between these parameters. This step will expedite our algorithm instead of using EM algorithm for each image. The performance of the method is reported based on its ability in correct segmentation of the test images. For evaluation of the proposed method, the manual segmentation of two observers was used as the gold standard.

For validation purpose, the mean signed and unsigned border positioning errors for each border were compared with other algorithms such as graph cut, *k*-means, and DP and the results are presented in Tables 1 and 2 for each boundary. We implemented each of these algorithms and tested them on our 90 two-dimensional test set. In *k*-means algorithm, we selected *k* = 3 and applied *k*-means algorithm on the image, directly. In graph cut method, we used the result of *k*-means algorithm to create initial prototype for each class and *C*_data_(*f*) was calculated by the distance between each image pixel to initial class made by *k*-means algorithm. *C*_smooth_(*f*) was calculated in the same method described in Section 2.3.3 by small change of selecting *r* = 3 which could give the best results. For DP method, the first step is similar to the proposed method in this paper (we applied boundary detection algorithm using dynamic programming to detect RPE layer boundary and eliminated the pixels above this boundary by making them equal to zero). Then we eliminated a region beneath the RPE (7 pixels below) and applied DP to search the image for another time. The results were based on the whitest route available after elimination of RPE layer.

According to Tables 1 and 2, the signed border positioning errors were 0.44 ± 1.18 pixels for BM extraction and 5.77 ± 2.77 pixels for choroid segmentation, and the unsigned border positioning errors were 2.48 ± 0.32 pixels for BM extraction and 9.79 ± 3.29 pixels for choroid segmentation, respectively. The errors between the proposed algorithm and the reference standard were similar to those computed between the observers. For example, the overall observer error was 2.64 ± 0.98 pixels and 8.78 ± 2.38 pixels for BM and CSI, respectively, which is comparable to the results of the algorithm. The border positioning errors of the proposed method showed significant improvement over other algorithms, compared in both of the tables.

To show the statistically significant improvement of the proposed method over the compared algorithms, Table 3 shows the obtained *P* values. The values confirm that our results have a significant improvement. For instance, the algorithm's overall *P* value against *k*-means and graph cut was less than 0.001 and against dynamic programming, it was less than 0.01.

**Figure 6:**
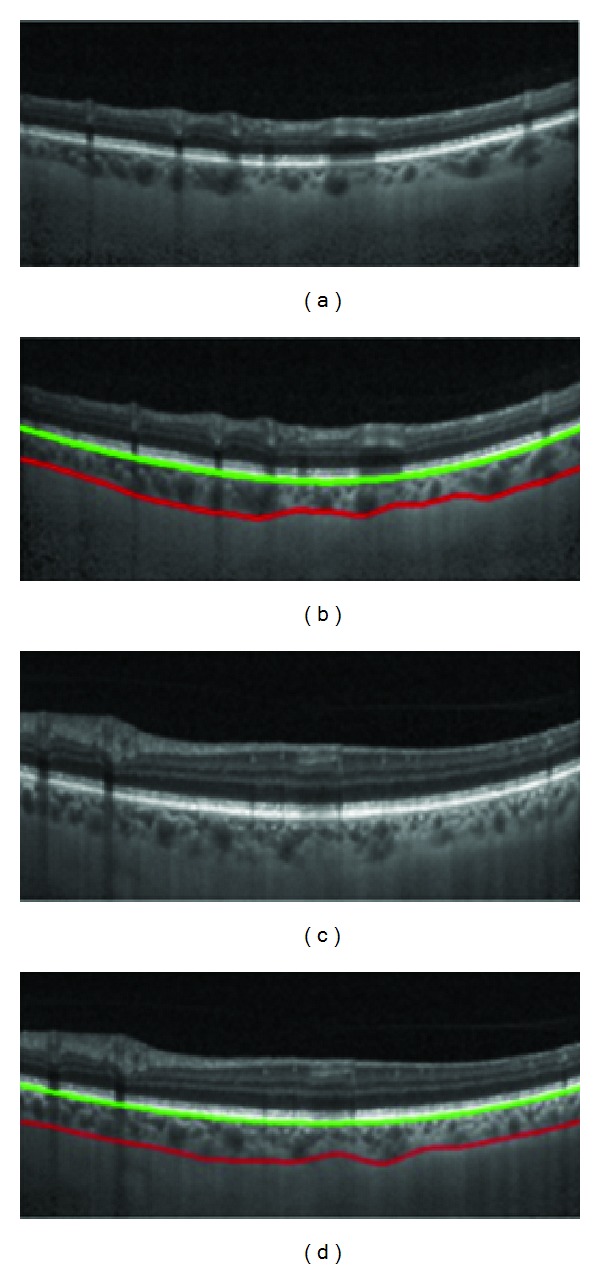
Two samples showing performance of the proposed method. The green line shows the BM boundary and the red line indicates the SCI boundary.

**Figure 7:**
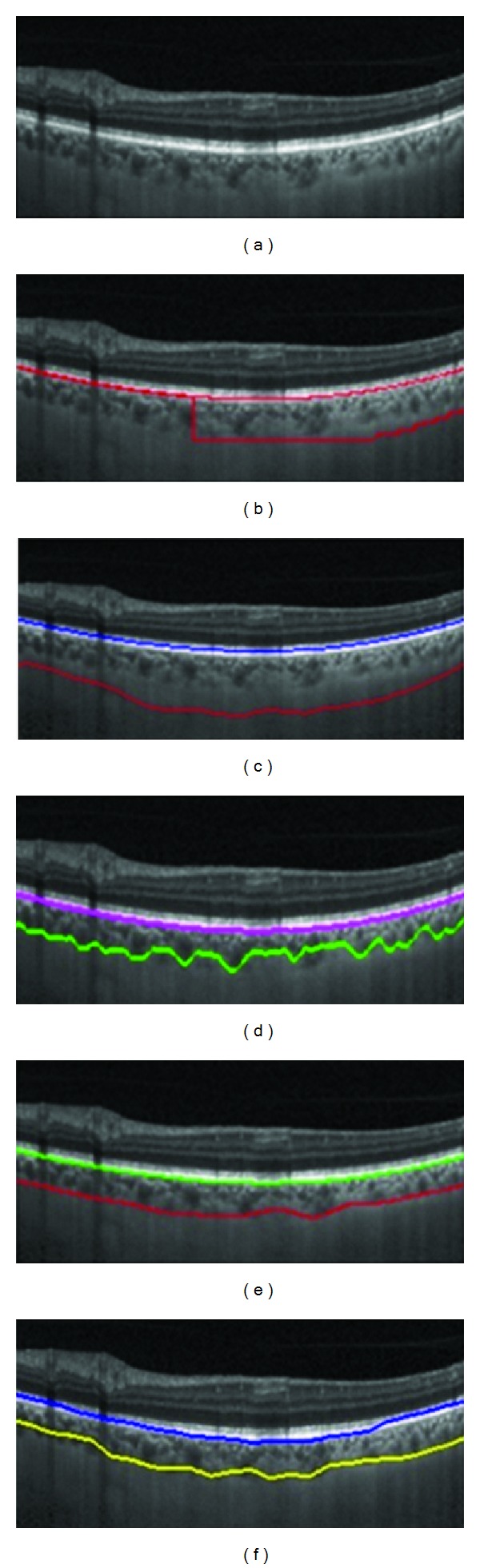
(a) EDI-OCT image, (b) graph cut result, (c) *k*-means result, (d) dynamic programming result, (e) our algorithm, and (f) manual segmentation.

The pixel resolution of our datasets in axial direction was 3.9 *μ*m/pixel. Therefore, mean signed positioning errors for localization of BM and choroid are 1.71 and 22.50 *μ*m, respectively. In accord with repeatability measurements for choroidal thickness of EDI-OCT images [12], it can be concluded that the positioning error has acceptable accuracy.  Figure 6 demonstrates two samples showing the performance of the proposed method. Furthermore, Figure 7 shows the results of segmentation using the proposed algorithm in comparison with other methods.

The computational complexity of the proposed algorithm is around 13 seconds in the case of using the saved parameters for constructed models; otherwise, if the computation consists of the model construction for one image, it takes around 21 seconds on a PC with Microsoft Windows XP x32 edition, Intel core 2 Duo CPU at 3.00 GHz, 4 GB RAM.

## 4. Conclusion

In this paper, a new method for choroid segmentation in EDI-OCT is introduced. In the first step, RPE boundary was segmented using DP method and the area above RPE was eliminated from next steps. Then, the wavelet descriptors were calculated for each pixel of the training images and assuming that the pdf of these descriptors are normal, their parameters were calculated to construct a model.

When a new test image is considered for segmentation, wavelet descriptors were calculated for each pixel and probability (*p*) of a pixel belonging to a particular class of the learned model was obtained. The value of probability (*p*) was then used in the construction of a graph cut segmentation.

The main limitation of this method is the need for manual segmentation to construct the model. Despite the fact that only a few training images are sufficient to produce good results, the manual labeling may be troublesome and replacing this step with an automatic method can be studied in future works. For example, in this work we choose several images as known data and extracted their mixture model's parameters for using as parameters of all new data in the database. For each database, arbitrary sample data can be used for constructing this mixture model (with known parameters) and then used for all data in database. However, the automatic version of this method can also been considered without using a predefined mixture model and by using EM algorithm for each image.

The new method has better accuracy than previous methods and the algorithm is tested on a larger dataset compared to older algorithms. The performance of the method is also fast and the implementation is relatively simple in comparison to more complicated algorithms.

As a future work, the proposed method should be tested on EDI images taken from other modalities to prove its robustness to the imaging technique. Furthermore, a 3D choroidal thickness map can be constructed using 3D OCT dataset which can assist the ophthalmologist in the diagnosis of the choroidal diseases. We also like to work on segmentation of the blood vessels in choroidal layer and produce a 3D vascular structure to give more information about distribution of the vessels.

## Figures and Tables

**Table 1:** Summary of mean signed border positioning errors (mean ± std).

	Avg. obs. versus our alg.	Avg. obs. versus graph cut alg.	Avg. obs. versus *k*-means alg.	Avg. obs. versus DP alg.	Obs. 1 versus obs. 2
BM	0.44 ± 1.18	4.30 ± 0.72	7.73 ± 3.35	0.44 ± 1.18	1.12 ± 1.29
Choroid	5.77 ± 2.77	57.69 ± 6.93	−31.25 ± 11.51	9.65 ± 5.41	7.65 ± 2.63

**Table 2:** Summary of mean unsigned border positioning errors (mean ± std).

	Avg. obs. versus our alg.	Avg. obs. versus graph cut alg.	Avg. obs. versus *k*-means alg.	Avg. obs. versus DP alg.	Obs. 1 versus obs. 2
BM	2.48 ± 0.32	4.51 ± 0.71	7.73 ± 3.35	2.48 ± 0.32	2.64 ± 0.98
Choroid	9.79 ± 3.29	65.69 ± 7.53	33.73 ± 12.23	12.48 ± 5.41	8.78 ± 2.38

**Table 3:** Improvement of the proposed method compared with other algorithms.

	*P* value, our alg. versus graph cut alg.	*P* value, our alg. versus *k*-means alg.	*P* value, our alg. versus DP alg.
BM	≪0.001	≪0.001	—
Choroid	≪0.001	≪0.001	≪0.01
